# Thalamic-limbic circuit dysfunction and white matter topological alteration in Parkinson’s disease are correlated with gait disturbance

**DOI:** 10.3389/fnagi.2024.1426754

**Published:** 2024-09-04

**Authors:** Qingguo Ren, Shuai Zhao, Rong Yu, Ziliang Xu, Shuangwu Liu, Bin Zhang, Qicai Sun, Qingjun Jiang, Cuiping Zhao, Xiangshui Meng

**Affiliations:** ^1^Department of Radiology, Qilu Hospital (Qingdao), Cheeloo College of Medicine, Shandong University, Qingdao, China; ^2^Medical Imaging and Engineering Intersection Key Laboratory of Qingdao, Qingdao, China; ^3^Shandong University of Traditional Chinese Medicine, Jinan, China; ^4^The First Affiliated Hospital of Air Force Military Medical University, Xi’an, China; ^5^School of Nursing and Rehabilitation, Cheeloo College of Medicine, Shandong University, Jinan, China; ^6^Department of Neurology, Qilu Hospital (Qingdao), Cheeloo College of Medicine, Shandong University, Qingdao, China; ^7^Department of Radiology, Xuecheng District People’s Hospital, Zaozhuang, China

**Keywords:** Parkinson’s disease, DTI—diffusion tensor imaging, freeing of gait, MRI, network neurodegeneration

## Abstract

**Background:**

Limbic structures have recently garnered increased attention in Parkinson’s disease (PD) research. This study aims to explore changes at the whole-brain level in the structural network, specifically the white matter fibres connecting the thalamus and limbic system, and their correlation with the clinical characteristics of patients with PD.

**Methods:**

Between December 2020 and November 2021, we prospectively enrolled 42 patients with PD and healthy controls at the movement disorder centre. All participants underwent diffusion tensor imaging (DTI), 3D T1-weighted imaging (3D-T1WI), and routine brain magnetic resonance imaging on a 3.0 T MR scanner. We employed the tract-based spatial statistical (TBSS) analytic approach, examined structural network properties, and conducted probabilistic fibre tractography to identify alterations in white matter pathways and the topological organisation associated with PD.

**Results:**

In patients with PD, significant changes were observed in the fibrous tracts of the prefrontal lobe, corpus callosum, and thalamus. Notably, the fibrous tracts in the prefrontal lobe and corpus callosum showed a moderate negative correlation with the Freezing of Gait Questionnaire (FOG-Q) scores (*r* = −0.423, *p* = 0.011). The hippocampus and orbitofrontal gyrus exhibited more fibre bundle parameter changes than other limbic structures. The mean streamline length between the thalamus and the orbitofrontal gyrus demonstrated a moderate negative correlation with Movement Disorder Society Unified Parkinson’s Disease Rating Scale (MDS-UPDRS) III (*r* = −0.435, *p* = 0.006). Topological parameters, including characteristic path length (*L*_p_), global efficiency (*E*_g_), normalised shortest path length (*λ*) and nodal local efficiency (*N*_le_), correlated moderately with the MDS-UPDRS, HAMA, MoCA, PDQ-39, and FOG-Q, respectively.

**Conclusion:**

DTI is a valuable tool for detecting changes in water molecule dispersion and the topological structure of the brain in patients with PD. The thalamus may play a significant role in the gait abnormalities observed in PD.

## Introduction

Parkinson’s disease (PD) is a chronic, progressive neurological disorder and the second most common neurodegenerative disease among middle-aged and elderly individuals (Samii et al., 2004). The thalamus is known to play a crucial role in motor regulation. Recent studies have highlighted the involvement of the central median para-fascicular (PF) complex of the thalamus in motor learning in patients with PD (Henderson et al., 2000). In addition to the PF complex, the thalamus has been a target for neurosurgical interventions in patients with PD with resting tremors for several years (Halliday, 2009). Furthermore, the limbic system is involved in the dominant pathophysiological processes of PD, as evidenced by early and substantial neuronal loss in many non-dopaminergic nuclei within the limbic regions (Sauerbier et al., 2016). It has been reported that activation of neurons in the accumbens nucleus projecting to the para-fascicular area can rescue depression-like phenotypes, and *de novo* parkinsonian patients with apathy exhibit bilateral microstructural alterations in the medial corticostriatal limbic system (Prange et al., 2019). Although both the thalamus and limbic system play significant roles in PD, the changes in their organisational microstructure are not yet fully understood.

Diffusion tensor imaging (DTI) allows for the noninvasive assessment of the tissue microstructure. The use of DTI in PD research has primarily focused on four areas: region of interest (ROI)-based studies, probabilistic fibre tractography, tract-based spatial statistics (TBSS), and white matter network topological analysis (Worker et al., 2014; Joshi et al., 2017; Li et al., 2017, 2018; Tan et al., 2019). Despite significant advancements in understanding the mechanisms of PD through DTI, current literature mainly comprises single-mode analyses focusing on specific structures. A systematic approach could uncover new mechanisms, such as studying the entire brain, thalamic, and limbic systems using multiple analytical methods (TBSS, probabilistic tracking, structural networks, etc.). Since the structural brain network is constructed based on macro fibres, analysing the microstructural alterations of white matter and their interactions with network impairments might provide deeper insights into the neural substrates of PD.

In this paper, we prospectively enrolled patients with sporadic PD at the movement disorder centre and age- and sex-matched healthy controls to identify changes in white matter pathways and topological organisation associated with PD. We investigated impairments in whole brain WM integrity in patients with PD using TBSS, further explored WM changes between the thalamic and limbic structures using probabilistic fibre tracking techniques, and finally analysed the global topological properties of the structural brain network. We hypothesise that the microstructure of white matter undergoes multidimensional changes and is associated with the clinical symptoms of the patients.

## Materials and methods

### Participants

This prospective clinical study was approved by the Ethics Committee of Qilu Hospital of Shandong University (Qingdao). We selected idiopathic PD outpatients from December 2020 to November 2021 at the movement disorder centre. All patients underwent a standardised clinical examination by a neurologist specialised in diagnosing movement disorders for over 10 years. The patients with PD were diagnosed according to the clinical criteria of the Movement Disorder Society (MDS) (Postuma et al., 2015). All patients exhibited bradykinesia and either resting tremor or rigidity, with a disease duration of about 4–7 years (5.69 ± 0.95, range 4–7). The severity of the disease for each patient was evaluated using the Movement Disorder Society Unified Parkinson’s Disease Rating Scale (MDS-UPDRS) in ON condition, Parkinson’s Disease Questionnaire 39 (PDQ 39), and Freezing of Gait Questionnaire (FOG-Q). The general cognitive abilities of all patients were assessed using the Chinese Version of the Mini-Mental State Examination (MMSE) and the Montreal Cognitive Assessment (MoCA). Psychological assessments were conducted using the Hamilton Anxiety Scale (HAMA) and the Hamilton Depression Scale (HAMD). Additionally, we enrolled age- and sex-matched healthy subjects during the same period as controls (HC), and their cognitive scales, anxiety and depression scales were also acquired. All participants signed informed consent forms.

### Exclusion criteria for the participants

Participants were excluded if they had acute cerebral infarction, defined as hyperintensity areas of brain parenchyma on diffusion-weighted imaging (DWI); lacunes, defined as hypo-intensity areas of brain parenchyma on T2 fluid-attenuated inversion recovery (T2FLAIR); brain tumours detected by CT or magnetic resonance imaging (MRI); or a history of neurological or psychiatric disorders. Ultimately, 42 patients with idiopathic PD (21 females/21 males, age = 63.59 ± 9.35 years) and 38 healthy controls (20 females/18 males, age = 62.26 ± 8.50 years) were included in our study. All subjects were right-handed, according to their self-reports.

### Scanning parameters

All MRI scans were performed on the same 3.0 T MRI scanner (Philips Ingenia, dStream brain special coil) in our hospital. Most of the patients (31 cases) were scanned on the same day after the scales assessment, the others were scanned in no more than 3 days before (6 cases) or after (4 cases) the scales assessment. The scanning range included the whole brain. T1 weighted 3D turbo gradient echo sequence was scanned as follows: FOV = 240 mm × 240 mm × 170 mm; voxel size = 1 mm × 1 mm × 1 mm; matrix = 240 × 240 × 170; NSA = 1; TR = 6.7 ms; TE = 3 ms; flip angle = 8°. For high directional spin-echo diffusion tensor imaging (DTI) the scanning parameters were as follows: FOV = 224 mm × 224 mm × 140 mm; voxel size = 2.0 mm × 2.0 mm × 2.0 mm; slice thickness = 2.0 mm; TR = 4,900 ms; TE = 95 ms; NSA = 2; reconstruction matrix = 112 × 112 × 70; flip angle = 90°; 32 diffusion directions with b = 1,000 s/mm^2^ and 0 s/mm^2^, and a total of 70 slices with no slice gap. For 2D FLAIR, the following parameters were used: TR = 7,000 ms, Flip Angle = 90°, TE = 125 ms, acquisition matrix = 272 × 176, and slice thickness = 6 mm. The MRI scan and clinical screening were performed within 2 days.

### Data pre-processing

The pre-processing of DTI images was performed using the FMRIB Software Library (FSL, http://fsl.fmrib.ox.ac.uk/). The processing procedures included the following: (1) brain extraction using the *bet* function, (2) correction for eddy currents and head motion using the *eddy_correction* function, and (3) computation of fractional anisotropy (FA), axial diffusivity (AD), mean diffusivity (MD), and radial diffusivity (RD) using the *dtifit* function.

### Tract-based spatial statistics

According to previous research (Zhang et al., 2022), TBSS was conducted using the *tbss* function in FSL, which involved four steps: (1) applying nonlinear registration of all FA images (or MD, AD, RD images) into standard space, (2) creating a mean FA image and skeletonising it, (3) projecting all subjects’ FA images (or MD, AD, RD images) onto the mean FA skeleton, and (4) performing statistical analysis.

### Probabilistic fibre tracking between thalamic and limbic structures

Probabilistic fibre tracking was conducted using FSL. Briefly, crossing fibres within each voxel of the brain were modelled using the *bedpostx* function. The fibres, weight, and burn-in parameters for *bedpostx* were chosen based on previous research (Behrens et al., 2007). Subsequently, probabilistic tracking with crossing fibres was performed using the *probtrackx* function. This step was executed in batch mode for individual nerves using the predefined ROIs. In this study, seven limbic ROIs—namely the anterior cingulate cortex, amygdala, orbitofrontal gyrus, hippocampus, posterior cingulate cortex, parahippocampal gyrus, and subcallosal gyrus—were selected to explore probabilistic fibre connectivity with the thalamus. Probabilistic fibre indices were calculated, including the number of streamlines, the mean streamline length, connectivity from the thalamus to the seven limbic ROIs, and the mean FA value and volumes of the thalamus linking to each limbic ROI.

### Cerebral structural network construction

In graph theory, a network is defined as a set of nodes connected by edges. Here, nodes and edges were defined as follows: the entire brain was divided into 90 regions using a prior grey matter (GM) atlas [automated anatomical labelling, AAL (Tzourio-Mazoyer et al., 2002)], each representing a network node. The edges were defined by probabilistic tractography (Behrens et al., 2007), and for each defined node, the connectivity probability was computed between that node and the remaining 89 nodes (Zhao et al., 2016).

Network topological properties analysis was performed using the GRETNA too, a graph theoretical network analysis toolbox for imaging connectomics (Wang et al., 2015). In this initial study, we focused on both global and node-level network characteristics (Wu et al., 2016). The global network properties included the following aspects: the mean clustering coefficient (*C*_p_), the characteristic path length (*L*_p_), the normalised *C*_p_ (*γ*), the normalised shortest path length (*λ*), the small-worldness (*ζ*), the global efficiency (*E*_g_), and the local efficiency (*E*_loc_). The nodal network included the following the nodal efficiency (*N*_e_), the nodal shortest path (*NL*_p_), the nodal clustering coefficient (*NC*_p_), and the nodal local efficiency (*NL*_e_). When *γ* ≫ 1 and *λ* ≈ 1, the brain network has the characteristics of a small-world (Zhong et al., 2017).

### Statistical analysis

The Statistical Package for the Social Sciences, version 22.0 (SPSS, Chicago, Illinois), was used to analyse the clinical data. Continuous variables were expressed as mean ± standard deviation and were analysed using the two-sample *t*-test. Categorical values were presented as counts and percentages and were analysed using the Pearson chi-square test. All statistical significance was defined as *p* < 0.05.

Threshold-free cluster enhancement correction was applied to the TBSS results. The significance of inter-group differences in probabilistic fibre indices, as well as global and nodal properties, were analysed using the two-sample *t*-test after controlling for age and gender. All graph theoretical parameter analyses were corrected using the Bonferroni method. Additionally, multiple linear regression analysis was employed to examine the associations between significant differences in structural network properties and probabilistic fibres and the severity of PD. The Spearman correlation was used to analyse these predictive features in relation to clinical factors. The outcomes were interpreted based on the degree of association as strong (|*r*| > 0.7), moderate (0.4 < |*r*| < 0.7), or mild (0.1 < |*r*| < 0.4), after considering significant correlation values (*p* < 0.05).

## Results

### Demographic characteristics

The clinical and demographic characteristics of the subjects are summarised in Table 1. There were no significant differences in age or sex between the two groups (*p* > 0.05). Similarly, no significant differences were observed between the two groups in the Mini-Mental State Examination (MMSE) and Montreal Cognitive Assessment (MoCA) scores (*p* > 0.05). However, significant differences were found in the Hamilton Anxiety Scale (HAMA) and Hamilton Depression Scale (HAMD), with higher scores observed in the PD group (*p* < 0.05).

**Table 1:** The clinical and demographic characteristics of the two groups.

	PD	HC	*p*
Age (y, mean ± SD, range)	63.59 ± 9.35 (43–83)	62.26 ± 8.50 (40–78)	0.514
Gender (male/female)	21/21	18/20	0.814
Education (y, mean ± SD, range)	8.26 ± 4.08 (0–15)	8.49 ± 4.09 (0–17)	0.808
Age of onset (y, mean ± SD, range)	57.33 ± 9.99 (35–78)	NA	
Hoehn–Yahr stage	1.87 ± 0.74 (1–4)	NA	
MDS-UPDRS	50.47 ± 24.39 (2–116)	NA	
MDS-UPDRS III	23.50 ± 12.70 (2–59)	NA	
MMSE	26.80 ± 4.11 (13–30)	28.00 ± 1.37 (25–30)	*0.953*
MoCA	23.24 ± 4.66 (9–30)	24.56 ± 3.11 (19–29)	0.170
HAMA	6.90 ± 4.81 (1–19)	3.37 ± 4.63 (0–22)	0.002
HAMD	12.02 ± 8.15 (0–32)	4.34 ± 4.37 (0–20)	*0.000*
PDSS	117.92 ± 24.91 (49–150)	NA	
PDQ-39	27.37 ± 23.47	NA	
FOG-Q	15.79 ± 15.65	NA	

MDS-UPDRS, I-IV, Movement disorder society unified Parkinson’s disease rating scale, Part I–Part IV; MMSE, Mini-Mental State Examination; MoCA, Montreal Cognitive Assessment; HAHA, Hamilton Anxiety Scale; HAMD, Hamilton Depression Scale; PDSS, Parkinson Disease Sleep Scale; PDQ 39, Parkinson’s Disease Questionnaire 39; FOG-Q, Freezing of Gait Questionnaire. The italicized *p*-value used a *U*-test and the others were *t*-test.

### TBSS and relations with clinical characteristics

**Figure 1:**
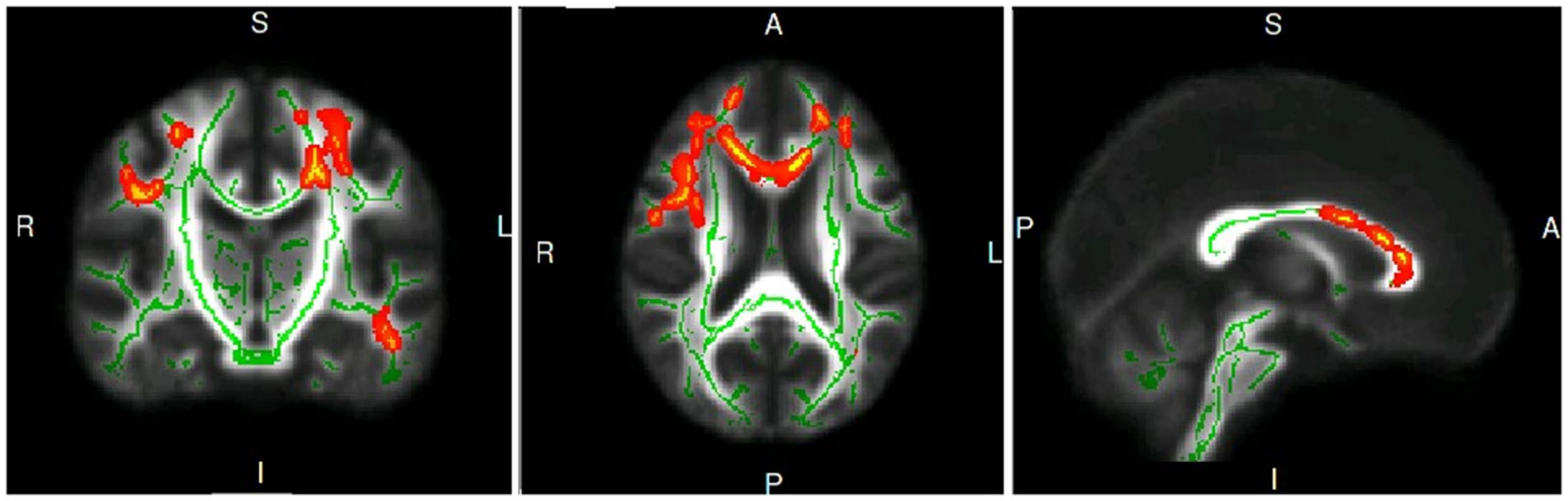
The coronal, axis and sagittal plane of white matter fiber clusters with statistical difference in PD group compared with healthy controls.

Patients with PD exhibited significantly lower FA in three clusters, as shown in Figure 1: Cluster 1 included the body of the corpus callosum (CC), genu of CC, bilateral anterior corona radiata, left superior corona radiata, and left posterior corona radiata; Cluster 2 included the right superior longitudinal fasciculus; Cluster 3 included the left posterior thalamic radiation. Cluster 1 had a mild negative correlation with the UPDRS III (*r* = −0.333, *p* = 0.041), UPDRS (*r* = −0.397, *p* = 0.014), and PDQ-39 (*r* = −0.345, *p* = 0.034), and a moderate negative correlation with the GFQ (*r* = −0.423, *p* = 0.011), as shown in Table 2.

**Table 2:** Correlation of the clusters and neuropsychological scales.

		Duration	MDS-UPDRS	MDS-UPDRS III	MMSE	MoCA	HAMA	HAMD	PDQ-39	FOG-Q	PDSS
Cluster 1	*r*	0.156	**−0.397**	**−0.333**	0.099	0.261	0.225	0.256	**−0.345**	**−0.423**	0.210
	*p*	0.325	**0.014**	**0.041**	0.535	0.118	0.157	0.116	**0.034**	**0.011**	0.200
Cluster 2	*r*	0.158	0.206	0.082	0.062	0.083	0.267	0.189	0.298	0.255	0.117
	*p*	0.318	0.215	0.623	0.695	0.625	0.091	0.248	0.069	0.139	0.479
Cluster 3	*r*	0.042	0.258	−0.29	0.065	0.018	0.087	0.048	0.245	0.132	0.094
	*p*	0.792	0.118	0.078	0.68	0.916	0.589	0.773	0.139	0.45	0.568

Bold black font R value is negative *p* < 0.05.

### Probabilistic fibre tracking between the thalamus and limbic region and their association with clinical characteristics

**Figure 2:**
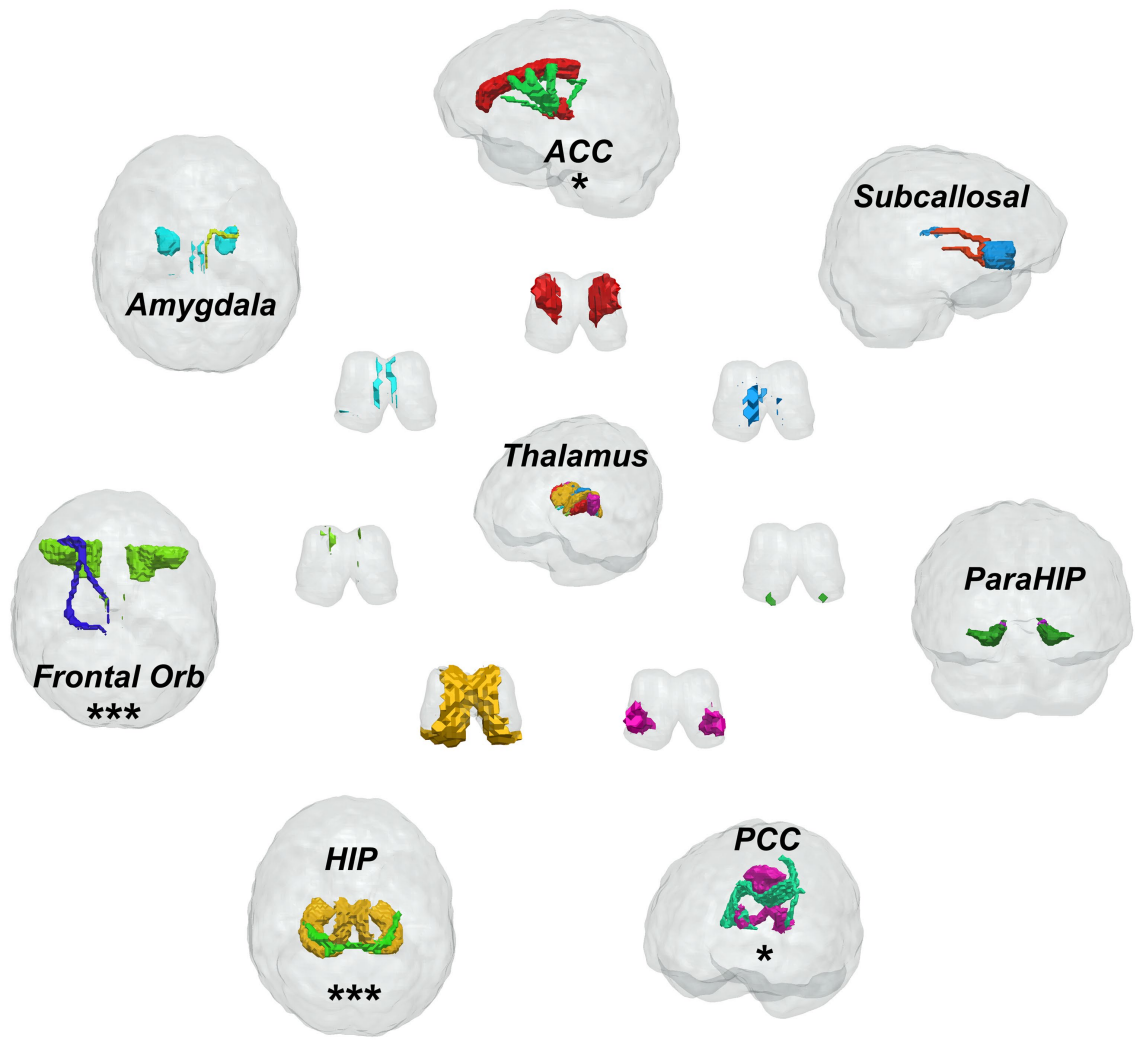
Fiber bundle construction of the thalamic-limbic system, one * presents one parameter has significant difference, three * means three parameters have significant difference between Parkinson’s disease patients and healthy controls.

**Figure 3:**
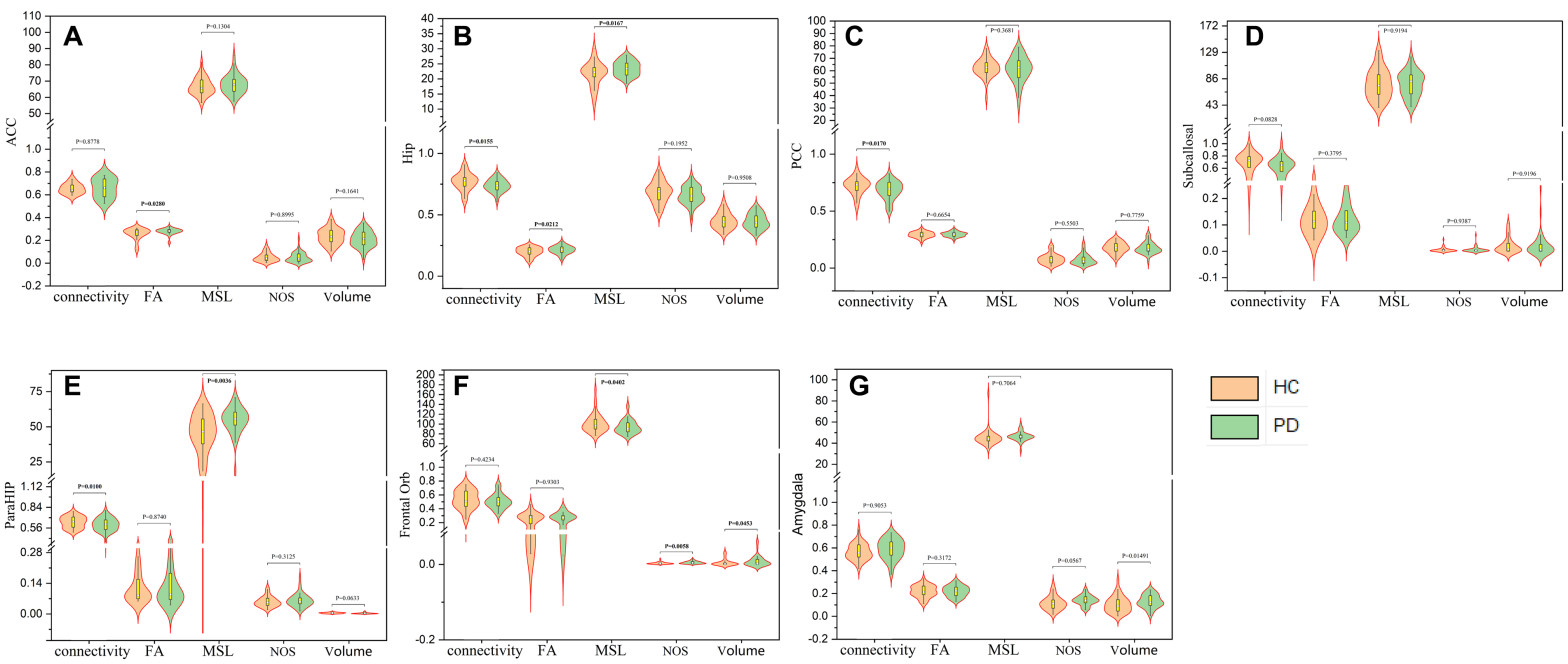
The five aspects of probabilistic fibres between the thalamus and anterior cingulate cortex **(A)**, the hippocampus **(B)**, the posterior cingulate cortex **(C)**, the subcallosal gyrus **(D)**, the parahippocampal gyrus **(E)**, the orbitofrontal gyrus **(F)** and the amygdala **(G)** respectively.

**Figure 4:**
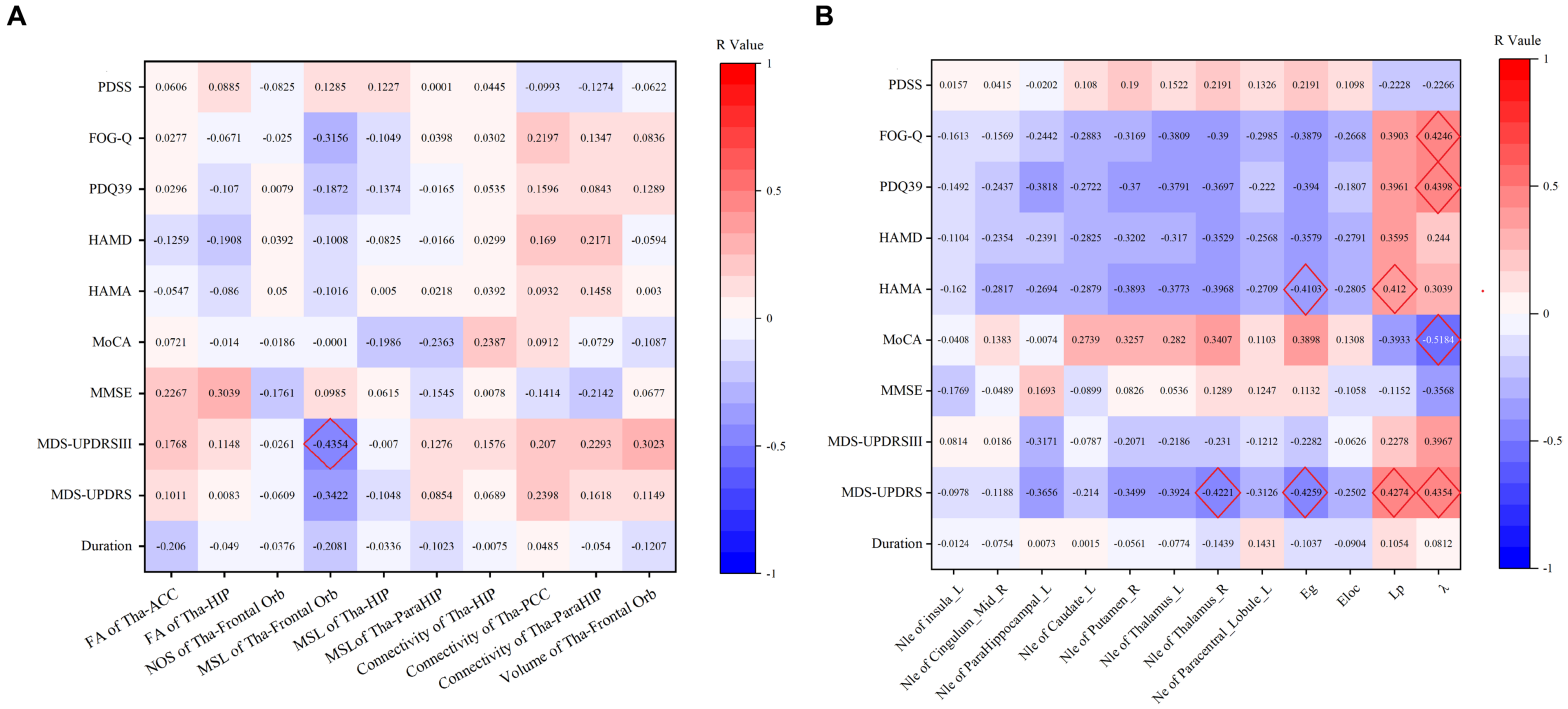
**(A)** Thalamic-limbic probabilistic fibers parameters correlation with neuropsychological scale. **(B)** Correlation of the TBSS positive clusters and neuropsychological scale.

Seven limbic regions including the anterior cingulate cortex, the amygdala, the orbitofrontal gyrus, the hippocampus, the posterior cingulate cortex, the parahippocampal gyrus, and the subcallosal gyrus were constructed, and the tracts between them and the thalamus were shown in Figure 2. We analysed five aspects of probabilistic fibres including: connectivity, FA, mean streamline length (MSL), the number of streamlines (NOS), and volume. Significant differences between the PD and HC groups were observed as follows: (1) the connectivity between the thalamus and the hippocampus decreased significantly in PD than HC (*p* = 0.0155, Figure 3B), the thalamus and the posterior cingulate cortex connectivity decreased significantly in PD than HC (*p* = 0.0170, Figure 3C), the thalamus and the parahippocampus connectivity decreased significantly in PD than HC (*p* = 0.0100, Figure 3E); (2) the mean FA values of the thalamus linked to the anterior cingulate cortex increased significantly in PD than HC (*p* = 0.0280, Figure 3A), the mean FA values of the thalamus linked to the hippocampus increased significantly in PD than HC (*p* = 0.0212, Figure 3B); (3) the mean streamline length (MSL) between the thalamus and the hippocampus increased significantly in PD than HC (*p* = 0.0167, Figure 3B), the thalamus and the parahippocampus MSL increased significantly in PD than HC (*p* = 0.0036, Figure 3E), while the thalamus and the orbitofrontal gyrus decreased significantly in PD than HC (*p* = 0.0402, Figure 3F); (4) the NOS between the thalamus and the orbitofrontal gyrus increased significantly in PD than HC (*p* = 0.0058, Figure 3F); (5) the volume of the thalamus linked to the orbitofrontal gyrus increased significantly in PD than HC (*p* = 0.0453, Figure 3F) Mean while, no significant differences were found in all the five aspects of probabilistic fibres between the thalamus and the subcallosal gyrus (*p* > 0.05, Figure 3D).

Further analysis revealed correlations between the probabilistic fibre parameters which showed statistical differences and the clinical characteristics, as outlined in Figure 4A. The MSL between the thalamus and orbitofrontal gyrus demonstrated: (1) a moderate negative correlation with the MDS-UPDRS III (*R* = −0.435, *p* = 0.006), (2) a mild negative correlation with the MDS-UPDRS (*R* = −0.342, *p* = 0.036). and (3) a mild negative correlation with the FOG-Q (*r* = −0.316, *p* = 0.065). The fractional anisotropy between the thalamus and hippocampus showed a mild positive correlation with the MMSE (*R* = 0.304, *p* = 0.053), and the volume between the thalamus and orbitofrontal gyrus had a mild positive correlation with the MDS-IPDR III (*R* = 0.302, *p* = 0.065).

### Network property calculation and correlation with clinical data

For global network properties, the *E*_loc_, *E*_g_, *L*_p_ and *λ* all showed significant differences between the PD and HC groups (*p* < 0.05), with significantly increased *L*_p_ and *λ* while decreased *E*_loc_ and *E*_g_ in PD group. Regarding nodal network properties, seven nodes, including the left insula, right median cingulate and paracingulate gyri, left parahippocampal gyrus, left caudate nucleus, right putamen, left thalamus, and right thalamus, demonstrated significantly decreased *N*_le_ in PD group comparing with HC group (*p* < 0.003). The *N*_e_ of the left paracentral lobule also showed a significant difference between the two groups as decreased in PD group (*p* < 0.0006). No significant differences were found in other parameters between the two groups, as shown in Table 3. We also calculated the betweenness centrality, community index, hierarchy, rich club, nodal cluster co-efficiency, nodal shortest path of the 90 nodes, there were no difference between the two groups, and the results were not given.

**Table 3:** Two-sample test results of the network properties between PD and HC.

	HC	PD	*p* Thrd	*p* vector	*T* Thrd	*T* vector
*C*_p_	0.0027 ± 0.001	0.0028 ± 0.001	0.05	0.5788	NA	0.5576
*L*_p_	50.2593 ± 4.134	53.253 ± 6.642	0.05	**0.0258**	2.2734	2.2734
*γ*	1.9066 ± 0.398	2.0309 ± 0.423	0.05	0.2363	NA	1.19
*λ*	1.2638 ± 0.029	1.2802 ± 0.036	0.05	**0.0377**	2.11	2.11
*ζ*	1.5041 ± 0.2842	1.5795 ± 0.277	0.05	0.3044	NA	1.03
*E*_g_	0.0200 ± 0.001	0.0190 ± 0.002	0.05	**0.0148**	2.4935	2.4935
*E*_loc_	0.0210 ± 0.002	0.0200 ± 0.001	0.05	**0.0034**	3.0281	3.0281
*N*_e_ 69	0.0179 ± 0.002	0.0160 ± 0.002	0.0006	**0.0001**	4.0067	−4.0067
*NL*_e_ 29	0.0206 ± 0.002	0.0192 ± 0.002	0.0030	**0.0014**	3.0839	−3.3212
*NL*_e_ 34	0.0193 ± 0.002	0.0178 ± 0.002	0.0030	**0.0006**	3.0839	−3.5995
*NL*_e_ 39	0.0196 ± 0.003	0.0178 ± 0.002	0.0030	**0.0026**	3.0839	−3.1085
*NL*_e_ 71	0.0200 ± 0.001	0.0188 ± 0.002	0.0030	**0.0024**	3.0839	−3.1415
*NL*_e_ 74	0.0199 ± 0.001	0.0187 ± 0.002	0.0030	**0.0026**	3.0839	−3.1195
*NL*_e_ 77	0.0201 ± 0.001	0.0189 ± 0.002	0.0030	**0.0028**	3.0839	−3.0839
*NL*_e_ 78	0.0200 ± 0.001	0.0187 ± 0.002	0.0030	**0.0023**	3.0839	−3.1589

29 insula_L, 34 Cingulum_Mid_R, 39 ParaHippocampal_L, 69 Paracentral_Lobule_L, 71 Caudate_L, 74 Putamen_R, 77 Thalamus_L, 78 Thalamus_R. Bold black font represents *p* < 0.05.

We further investigated the correlation of these properties with clinical test results, and moderate correlations were noted as follows: (1) *λ* showed a moderate negative correlation with MoCA (*R* = −0.5184, *p* = 0.0012), a moderate positive correlation with MDS-UPDRS (*R* = 0.4354, *p* = 0.0063), with PDQ39 (*R* = 0.4398, *p* = 0.0073), with FOG-Q (*R* = 0.4246, *p* = 0.011), and a nearly moderate positive correlation with MDS-UPDRS III (*R* = 0.3967, *p* = 0.0137); (2) *L*_p_ exhibited a moderate positive correlation with MDS-UPDRS (*R* = 0.4274, *p* = 0.0074), with HAMA (*R* = 0.412, *p* = 0.0074), and a nearly moderate positive correlation with PDQ39 (*R* = 0.3961, *p* = 0.0168) and with FOG-Q (*R* = 0.3903, *p* = 0.0205), and a nearly moderate negative correlation with MoCA (*R* = -0.3933, *p* = 0.0177); (3) *E*_g_ showed a moderate negative correlation with MDS-UPDRS (*R* = −0.4259, *p* = 0.0077), and with HAMA (*R* = −0.4103, *p* = 0.0077), and a nearly moderate negative correlation with PDQ-39 (*R* = −0.394, *p* = 0.0174); (4) *N*_le_ of the right thalamus showed a moderate negative correlation with MDS-UPDRS (*R* = −0.4221, *p* = 0.0083), a nearly moderate negative correlations with *FOG-Q* (*R* = −0.39, *p* = 0.0206) and with HAMA (*R* = −0.3968, *p* = 0.0102); (5) *N*_le_ of the left thalamus showed a nearly moderate negative correlation with MDS-UPDRS (*R* = −0.3924, *p* = 0.0148). These results are detailed in Figure 4B.

## Discussion

In this study, we investigated the changes in WM fibres between the thalamus and limbic structures based on DTI in patients with PD compared to healthy controls, as well as the whole-brain level of structural networks, structural impairments of WM, and the relationships between fibre cluster features, network parameters, and clinical characteristics of patients with PD. Our findings are threefold: first, fibrous tracts of the prefrontal lobe, corpus callosum, and thalamus were the primary fibre clusters altered in patients with PD, showing a moderate negative correlation with the GFQ; second, the hippocampus and orbitofrontal gyrus exhibited more changes in fibre bundle parameters than other limbic structures, with the mean streamline length between the thalamus and orbitofrontal gyrus displaying a moderate negative correlation with the MDS-UPDRS III; third, nodal local efficiency proved to be a sensitive index for differentiating PD from healthy controls, particularly focusing on the basal ganglia area, and parameters including the characteristic *L*_p_, *E*_g_, *λ* and *N*_le_ showed moderate correlations with MDS-UPDRS, MoCA, HAMA, PDQ 39, and FOG-Q.

White matter tract abnormalities in PD have been extensively reported, revealing significant abnormalities. Lower FA in patients with PD was observed in a broad area, including the corpus callosum (CC) and internal and external capsules, particularly in groups with severe symptoms, psychosis or cognitive impairments, and freezing of gait (Goldman et al., 2017; Guimarães et al., 2018; Pietracupa et al., 2018; Lenka et al., 2020). However, previous results have been inconsistent; a meta-analytical approach indicated that FA reduction was identified predominantly in the body of the CC and the left inferior fronto-occipital fasciculus (Wei et al., 2021). Another study using multiple regression analysis revealed that the severity of the akinetic-rigid subtype was negatively associated with callosal FA at the baseline and 24-month follow-up, particularly in the anterior portion of the CC (Amandola et al., 2022). In our results, the prefrontal lobe fibrous tracts, including the CC, showed lower FA in the PD groups and had a moderate negative correlation with the FOG-Q and a mild negative correlation with the MDS-UPDRS, aligning with the results of previous studies (Pietracupa et al., 2018). The CC facilitates the communication of sensory, motor, and cognitive information between cerebral hemispheres. The MDS-UPDRS reflects the severity of the disease, correlating the FA of the CC with motor symptoms of PD. Unlike previous studies, our results did not show an apparent correlation of FA in the three clusters with cognition, possibly because areas other than the CC play a leading role. Indeed, patients with PD without corpus callosum involvement suggest that bilateral degenerative changes occur independently of the interhemispheric connections (Kho et al., 2019), which could also explain our findings to some extent. We conjecture that callosal microstructure alterations in the anterior CC may serve as a viable biomarker for gait abnormalities as indicated by the FOG-Q and is a sensitive area with high reliability, potentially targeting neural regulation therapies such as transcranial magnetic stimulation therapy.

The thalamus serves a more significant role than merely relaying information; motor thalamic neurons dynamically alter information received from cortical regions (Halliday, 2009). In recent years, the midline nuclei of the thalamus have garnered significant attention as substrates for cognitive functions, as well as mood, emotions, and reward processing. The dorsal thalamic midline, including the paraventricular thalamic (PVT) nucleus, receives multiple inputs from the brainstem and hypothalamus and targets the medial prefrontal cortex, accumbens nucleus, and amygdala (Kaitz and Robertson, 1981; Wolff et al., 2015). The anterior thalamic nuclei, integrated with the hippocampus, mammillary bodies, and cingulate region, play roles in spatial and nonspatial cognition (Colavito et al., 2015). However, until recently, the associated functions of the thalamus and limbic system have received relatively little attention in the pathology of PD. Our results indicated that the hippocampus and orbitofrontal gyrus, linked to the thalamus, changed more significantly than other limbic structures, which may relate to the patients’ non-motor disorders. In addition to non-motor disorders, our findings revealed that the mean streamline length between thalamus and the orbitofrontal gyrus had a moderate negative correlation with the MDS-UPDRS III. Previously, it has been reported that patients with PD exhibit abnormal task-related activity during both reward anticipation in the ventral striatum and reward outcome in the orbitofrontal cortex (du Plessis et al., 2018). More of that, the mean streamline length between thalamus and the orbitofrontal gyrus decreased in PD group and had a mild negative correlation with the MDS-UPDRS, a mild positive correlation with the FOG-Q. The fractional anisotropy between the thalamus and hippocampus showed a mild positive correlation with the MMSE, and the volume between the thalamus and orbitofrontal gyrus increased in PD and had a mild positive correlation with the MDS-IPDR III. Estimating damaged streamlines contribute our knowledge about the extent of the PD structural pathology. Thus, the orbitofrontal gyrus might be a potential target for treating motor symptoms in PD.

The efficiency of network information transmission was decreased in patients with PD, and *E*_g_ was moderately correlated with the MDS-UPDRS, HAMA and the PDQ-39. Previously, abnormalities in global network properties were also found in dyskinetic patients with PD compared to non-dyskinetic ones, and increased *NL*_p_ in the bilateral inferior frontal gyrus (IFG), right putamen, and right thalamus was observed in dyskinetic PD (Wang et al., 2019). Our results showed that most of the structural network parameters correlated with motor symptoms, especially in the thalamus. Additionally, *E*_loc_, *E*_g_, *L*_p_, and *λ* exhibited significant differences between PD and HC groups; *N*_le_ and *N*_e_ also showed significant differences between the two groups. The *λ* showed a moderate negative correlation with MoCA, a moderate positive correlation with MDS-UPDRS, with PDQ39, with FOG-Q, and with MDS-UPDRS III; The *L*_p_ exhibited a moderate positive correlation with MDS-UPDRS, with HAMA, with PDQ39 and with FOG-Q, a nearly moderate negative correlation with MoCA. The *E*_g_ showed a moderate negative correlation with MDS-UPDRS, with HAMA and a nearly moderate negative correlation with PDQ-39. Consistent with the results of previous studies, deterministic tractography of PD patients’ brain structural networks showed a statistically significant decrease in *E*_g_ (Li et al., 2017; Wen et al., 2017). Furthermore, significantly decreased *N*_le_ was previously reported in patients with PD compared to HCs (Wang et al., 2020). Moreover, the network parameters had a moderate correlation with both motor and non-motor symptoms. We concluded that *λ*, *L*_p_, *E*_g_, *N*_e_, and *N*_le_ are sensitive indices for differentiating PD from HC, and correlating with the motor symptoms, especially with freeing of gait of PD patients, that means the decreasing of information transmission efficiency is the main cause of motor symptoms in Parkinson’s disease, and the thalamus plays a crucial role in the development of PD which is consistent with the pathological process of PD.

Our study had some limitations. First, we included patients with a disease duration of 4–5 years, some of whom were already in the advanced stages of the disease. Patients in these stages are more likely to experience changes in brain microstructure. Second, this was a small sample pilot study. Future research should include patients in the early stages of the disease and involve a larger sample size.

In conclusion, diffusion tensor imaging is a valuable method for detecting alterations in white matter fibres and the global structural, topological organisation in Parkinson’s disease. The hippocampus and orbitofrontal gyrus showed significant variations compared to other limbic structures, and the fibrous tracts of the prefrontal lobe and corpus callosum might play a role in the pathogenesis of motor dysfunction in PD; the global and nodal local efficiency contributed to the functional deficits. These findings suggest that the hippocampus, orbitofrontal gyrus, prefrontal lobe, and corpus callosum could be potential targets for modulation in PD. DTI could help us understand the mechanisms underlying PD symptoms. More research is needed in the future.

## Data availability statement

The datasets presented in this study can be found in online repositories. The names of the repository/repositories and accession number(s) can be found at: Picture Archiving and Communication System in Qilu Hospital of Shandong University (Qingdao).

## Ethics statement

The studies involving humans were approved by Qilu Hospital of Shandong University (Qingdao). The studies were conducted in accordance with the local legislation and institutional requirements. The participants provided their written informed consent to participate in this study.

## Author contributions

QR: Formal analysis, Investigation, Methodology, Funding acquisition, Writing – original draft. SZ: Data curation, Investigation, Writing – review & editing. RY: Conceptualization, Resources, Writing – review & editing. ZX: Formal analysis, Methodology, Software, Writing – review & editing. SL: Methodology, Writing – review & editing. BZ: Data curation, Resources, Visualization, Writing – review & editing. QS: Data curation, Writing – review & editing. QJ: Conceptualization, Investigation, Writing – review & editing. CZ: Conceptualization, Funding acquisition, Writing – review & editing. XM: Conceptualization, Funding acquisition, Writing – review & editing.
